# Correlation between tumor infiltrating immune cells and peripheral regulatory T cell determined using methylation analyses and its prognostic significance in resected gastric cancer

**DOI:** 10.1371/journal.pone.0252480

**Published:** 2021-06-04

**Authors:** Koung Jin Suh, Jin Won Kim, Ji Eun Kim, Ji Hea Sung, Jiwon Koh, Kui-Jin Kim, Ji-Won Kim, Sang-Hoon Ahn, Do Joong Park, Hyung-Ho Kim, Hye Seung Lee, Keun-Wook Lee

**Affiliations:** 1 Department of Internal Medicine, Seoul National University Bundang Hospital, Seoul National University College of Medicine, Seongnam, Republic of Korea; 2 Yonsei Biomedical Research Institute, College of Medicine, Yonsei University, Seoul, Republic of Korea; 3 Department of Pathology, Seoul National University Hospital, Seoul National University College of Medicine, Seoul, Republic of Korea; 4 Biomedical Research Institute, Seoul National University Bundang Hospital, Seongnam, Republic of Korea; 5 Department of Surgery, Seoul National University Bundang Hospital, Seoul National University College of Medicine, Seongnam, Republic of Korea; 6 Department of Surgery, Seoul National University Hospital, Seoul National University College of Medicine, Seoul, Republic of Korea; 7 Department of Pathology, Seoul National University Bundang Hospital, Seoul National University College of Medicine, Seongnam, Republic of Korea; Baylor College of Medicine, UNITED STATES

## Abstract

Peripheral regulatory T cells (pTregs) are a highly immunosuppressive fraction of CD4+ T cells. We aimed to evaluate the clinical significance of pTregs in patients with gastric cancer and to determine the correlation between pTregs and immune cell infiltration in tumor microenvironment. pTregs status was determined by assessing the pTreg/total T-cell ratio (ratio of Foxp3 Treg-specific demethylated region (TSDR) to CD3G/CD3D demethylation, so-called Cellular Ratio of Immune Tolerance “ImmunoCRIT”) using methylation analyses in 433 patients with gastric cancer who received curative surgery. Among 422 evaluable patients, 230 (54.5%) had high ImmunoCRIT (> 21.0). Patients with high ImmunoCRIT had significantly shorter disease-free survival (DFS) and overall survival (OS) than those with high ImmunoCRIT (*p* = 0.030, *p* = 0.008, respectively). In multivariate analysis, high ImmunoCRIT kept a prognostic role for shorter OS (hazard ratio [HR] 1.9, 95% confidence interval [CI] 1.4–2.9; *p* = 0.005). CD3+ cell density and CD4+ cell density was significantly higher within the tumor in high ImmunoCRIT group than those in low ImmunoCRIT group (CD3+ cell, 202.12/mm^2^ *vs*. 172.2/mm^2^, *p* = 0.029; CD4+ cell, 56.5/mm^2^ *vs*. 43.5/mm^2^, *p* = 0.007). In conclusion, the peripheral ImmunoCRIT determined by epigenetic methylation analysis provides prognostic information in resected gastric tumors.

## Introduction

Tumor microenvironment (TME) is the cellular environment where tumor exists, including the surrounding blood vessels, immune cells, fibroblasts, myeloid-derived suppressor cells (MDSC), lymphocytes, signaling ligands, and extracellular matrix [1]. In TME, interactions between tumor and immune cells are known to play a crucial role at all stages of carcinogenesis, and distinct classes and subclasses of tumor immune microenvironment (TIME) have been shown to be associated with response to immunotherapeutics as well as with prognosis [2]. In the era of immunotherapy, deeper analyses of TIME may be helpful in identifying patient populations who are responsive to a cancer immunotherapy.

Despite this clinical relevance, assessing TIME is challenging. The composition, function, and location of immune cells within the TIME can change over time during the cancer development [2]. TIME can also change under treatment pressure, and TIME in metastasis is known to be different from that of the primary tumor [3]. However, repetitive and multiple acquisitions of tumor tissues from cancer patients for TIME assessment are not generally feasible. Therefore, easier methods for assessing TIME are needed.

Since TIME is part of the host immunity, efforts have been made to identify peripheral blood markers to estimate the composition of immune cells in TIME. We have previously reported that the neutrophil-to-lymphocyte ratio (NLR) and prognostic nutritional index (PNI) were associated with the density of CD4+ immune cells in TIME, which leads to prognostic values of systemic inflammation in gastric cancer [4]. Other peripheral blood markers such as circulating MDSC and peripheral regulatory T cells (pTregs) have also been considered as liquid biomarkers. Lower frequencies of MDSC were associated with better clinical response to ipilimumab treatment in patients with melanoma [5]. Patients with higher pTregs levels had poor clinical outcomes in follicular lymphomas, ovarian cancer, and non-small-cell lung cancer [6–8]. Therefore, these peripheral blood biomarkers may be associated with TIME and provide prognostic information.

Recent advancement in epigenetic analysis allows the assessment of Tregs/total CD3+ T-lymphocytes (tTL) ratio in long-term stored tumor tissues and blood samples. The Tregs-to-tTL ratio by epigenetic analysis, so-called Cellular Ratio of Immune Tolerance (“ImmunoCRIT”), is thought to be an important determinant of immune tolerance and tended to increase in tumor biopsies as compared to benign tissues and gradually augmented strictly depending on tumor aggressiveness [9]. Peripheral blood ImmunoCRIT of initially healthy participants was reported as an independent risk factor for lung, colorectal, and ER-negative breast cancer [10].

Therefore, this study aimed to evaluate the clinical significance of peripheral ImmunoCRIT in patients with resected gastric cancer and to determine the correlation between ImmunoCRIT and specific immune cell infiltration in TIME as an underlying mechanism.

## Materials and methods

### Study population

A total of 433 patients with gastric cancer who underwent curative-intent resection between 2005 and 2013 at Seoul National University Bundang Hospital (SNUBH) were eligible for this study. Clinical data were retrieved from patient medical records. Adjuvant chemotherapy was administered as per physician’s discretion. The median follow-up duration was 83.1 (range, 3.1–141.0) months. This study was approved by the Institutional Review Board of SNUBH (B-1603/338-304) and was conducted in accordance with the Declaration of Helsinki. All samples and records were deidentified and anonymized prior to the study. Informed consent was obtained from all subjects. No individual-level data are reported.

### Measurement of peripheral blood Tregs by methylation assay

#### Pyrosequencing analysis

We used the bisulfite pyrosequenicng method for methylation analyses of FOXP3-TSDR and CD3D/CD3G genes after sodium bisulfite modification (S1 Method). The MS-qPCR (methylation-specific—quantitative polymerase chain reaction) of FOXP3-TSDR [11, 12] and the bisulfite-sequence assay of CD3D/CD3G [13] have been adopted as previously described (S1 Method). The primer sequence is listed in S1 Table. S1 Fig shows an example of two pyrograms analyzing 4 or 6 CpG sites of FOXP3-TSDR and CD3D/CD3G genes. The methylation percentage was calculated based on the average degree of methylation at 4 or 6 CpG sites formulated in pyrosequencing (S1 Method).

To validate these epigenetic patterns of FOXP3-TSDR and CD3D/CD3G by pyrosequencing analysis to identify corresponding immune cell density, live immune cells were sorted from fresh blood of the male corresponding author, including CD8+, CD15+, and CD4+/CD25+ immune cells. Different epigenetic patterns according to immune cells were demonstrated (S2 Fig).

#### The cellular ratio of immune tolerance (ImmunoCRIT)

ImmunoCRIT was used as an important determinant of immune tolerance and was defined as the pTregs-to-tTL ratio determined according to the FoxP3-TSDR-to-CD3G/CD3D demethylation ratio. For female patients, this rate was corrected by a factor of 2 because one of the two TSDR alleles is methylated as a result of X inactivation [9, 10]. The cutoff value (21.0) of ImmunoCRIT for survival was determined by finding the optimal cut point for continuous covariates with time-to-event outcomes [14].

#### Immunohistochemical analysis of immune cells in the TIME

Tumor microarray construction and antibody information shows in S1 Method. The number of arginase+, CD68+, CD163+ immune cells, and densities of CD3+, CD4+, CD8+, CD45RO+, and FoxP3+ immune cells in the TME were assessed using a previously described image analysis system (S1 Method) [15]. All immune cells’ markers were evaluated separately within the tumor central and peripheral areas. Immune cell density was defined as the number of strong positive cells (3+) per unit of tissue surface area (CD3, CD4, CD8, Foxp3, CD45RO-cells/mm^2^; CD68, CD163, arginase-pixels/mm^2^).

#### Systemic inflammation markers

The NLR and PNI were used as markers of systemic inflammation. NLR was calculated as the ratio of neutrophil count to the lymphocyte count, and PNI was calculated as previously described: 10 × albumin concentration (g/dL) + 0.005 × total lymphocyte count (/mm^3^). The cutoff value for NLR was defined using the receiver operating characteristic curve for survival and the value of 2.5, with 27.9% sensitivity and 85.3% specificity (Area under the curve = 0.571, 95% CI 0.508–0.635). The cut-off value to define PNI elevation was 54, which was a median value of PNI in this study population.

### Statistical analysis

The baseline characteristics of patients and clinicopathological findings according to ImmunoCRIT were evaluated using the Pearson chi-square test. DFS and OS from the date of surgical resection were calculated using the Kaplan–Meier method, and values were compared using the log-rank test. Univariate Cox proportional-hazard regression (PHR) analyses were performed to evaluate the predictive values of each variable, and variables found to be significantly predictive in the univariate analyses were introduced into a multivariable Cox PHR model with backward elimination methods using *P*-value < 0.05 as entry criterion, and *P*-value ≥ 0.10 as removal criterion for DFS and OS. All tests were two-sided, and *P*-value of <0.05 was considered significant. All analyses were performed using the SPSS software version 21 (Chicago, IL, USA) and R Statistical Software (R version 3.4.4).

## Results

### Patient characteristics

Among the total of 433 patients enrolled, blood samples of 11 patients did not pass the quality control process for ImmunoCRIT. Finally, 422 patients were analyzed. Their clinical characteristics are shown in Table 1. The median age was 59 (range, 20–87) years, and 274 patients were men (64.9%). Most patients had subtotal gastrectomy (61.8%). Diffuse type by Lauren classification was the most frequently identified subtype (54.0%). Lymphatic, vascular, and perineural invasions were found in 69.8%, 16.2%, and 65.6% of patients, respectively. The percentages of cancers according to stages were as follows: 48.1% for stage II (n = 203) and 51.9% for stage III (n = 219). High NLR and low PNI that indicate worse prognosis were identified in 19.4% and 47.2% of patients, respectively. All patients, except one who did not take the prescribed TS-1, received adjuvant chemotherapy such as TS-1 (85.5%) or fluoropyrimidine combined with platinum (14.2%).

**Table 1:** Baseline characteristics according to peripheral ImmunoCRIT.

Variables			ImmunoCRIT		*p-*value
		Total (N = 422)	High (N = 230)	Low (N = 192)	
Age (years)	< 70	342 (81.0)	182 (53.2)	160 (46.8)	0.273
	≥ 70	80 (19.0)	48 (60.0)	32 (40.0)	
Sex	Male	274 (64.9)	87 (31.8)	187 (68.2)	<0.001
	Female	148 (35.1)	143 (96.6)	5 (3.4)	
Lauren classification	Intestinal	154 (36.5)	67 (43.5)	87 (56.5)	0.003
	Diffuse	228 (54.0)	140 (61.4)	88 (38.6)	
	Mixed	36 (8.5)	19 (52.8)	17 (47.2)	
Stage by AJCC 7th	II	203 (48.1)	101 (49.8)	102 (50.2)	0.059
	III	219 (51.9)	129 (58.9)	90 (41.1)	
NLR	Low (<2.5)	331 (78.4)	169 (51.1)	162 (48.9)	0.016
	High (≥2.5)	82 (19.4)	54 (65.9)	28 (34.1)	
PNI	High (≥54)	189 (44.8)	121 (64.0)	68 (36.0)	<0.001
	Low (< 54)	199 (47.2)	90 (45.2)	109 (54.8)	
Adjuvant chemotherapy	No adjuvant	1 (0.2)	1 (100.0)	0 (0.0)	0.312
	TS-1	361 (85.5)	196 (54.3)	165 (45.7)	
	Capecitabine + oxaliplatin	40 (9.5)	19 (47.5)	21 (52.5)	
	Fluorouracil + cisplatin	20 (4.7)	14 (70.0)	6 (30.0)	

AJCC, American Joint Committee on Cancer; NLR, neutrophil to lymphocyte ratio; PNI, prognostic nutritional index; WHO, world health organization; AJCC, American Joint Committee on Cancer.

### The clinicopathological correlation according to peripheral ImmunoCRIT

**Fig 1:**
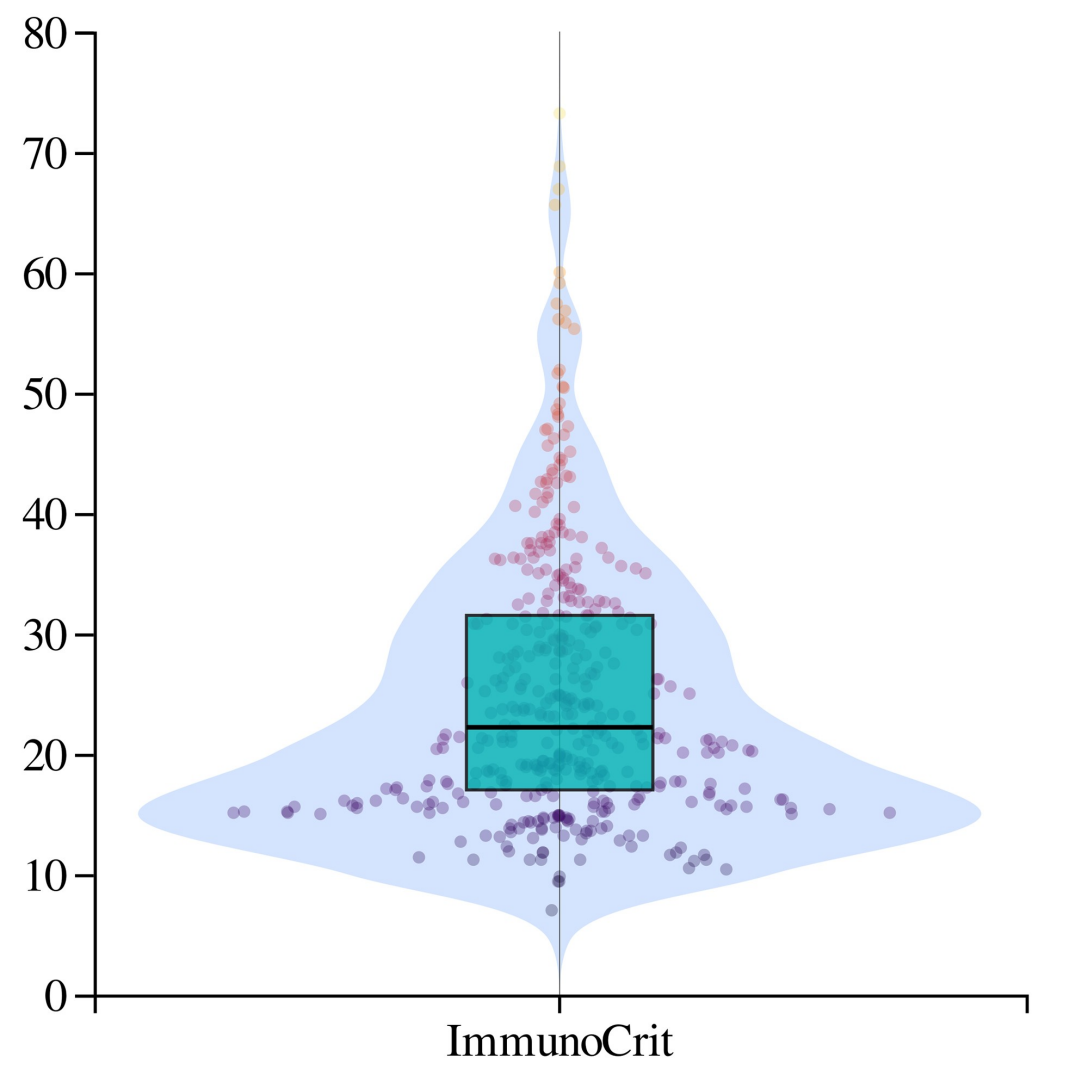
Violin plot with dot of the ImmunoCRIT.

The median values of FoxP3 Treg-specific demethylated region (TSDR) and CD3G/CD3D demethylation were 6.7% and 32.3%, respectively. The clinicopathological correlation according to the level of FoxP3-TSDR is shown in S2 Table. The distribution of ImmunoCRIT is showed in Fig 1. The median ImmunoCRIT was 21.9 (range, 7.0–73.2). Using the cut-off value of 21.0 for peripheral ImmunoCRIT, 54.5% (n = 230) were classified as having high ImmunoCRIT and 45.5% (n = 192) of patients were classified as having low ImmunoCRIT. No significant difference was observed according to ImmunoCRIT in age, stage, and adjuvant chemotherapy (Table 1). NLR and PNI were significantly correlated with ImmunoCRIT (*p* = 0.016 and *p*<0.001, respectively).

### Prognostic significance of peripheral ImmunoCRIT

**Fig 2:**
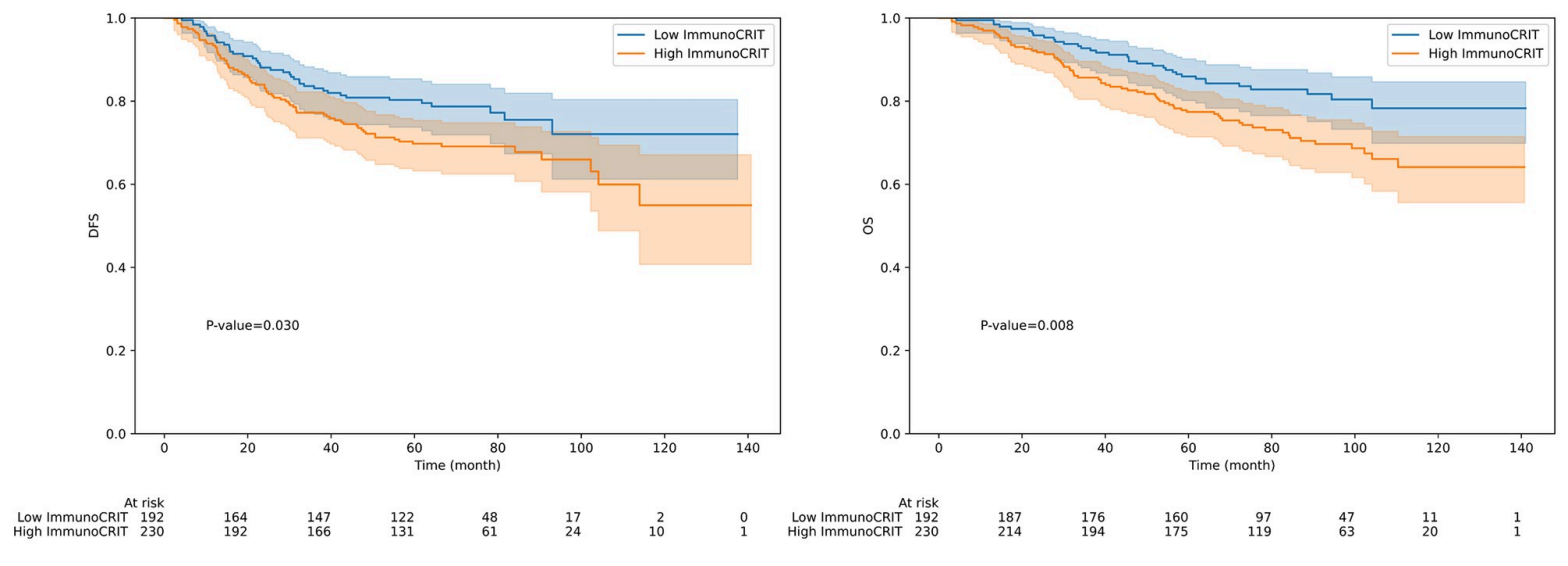
(a) Disease-free survival (DFS) and (b) Overall survival (OS) according to the ImmunoCRIT.

Patients with low peripheral ImmunoCRIT had significantly longer disease-free survival (DFS) and overall survival (OS) than those with high ImmunoCRIT (*p* = 0.030, *p* = 0.008, respectively; Fig 2). The 5-year DFS rate was 79.6% in the low ImmunoCRIT group and 69.8% in the high ImmunoCRIT group. In multivariate analysis that included peripheral ImmunoCRIT, age, lymphatic invasion, vascular invasion, perineural invasion, stage, NLR, and PNI as covariables, vascular invasion, perineural invasion and stage III remained as independent predictors of poorer DFS (Table 2). The 5-year OS rate was 85.9% in the low peripheral ImmunoCRIT group and 77.4% in the high ImmunoCRIT group. In multivariate analysis, high peripheral ImmunoCRIT was an independent predictor of shorter OS (HR = 1.9, 95% CI 1.2–2.9, *p* = 0.005) with vascular invasion, perineural invasion, and stage III (Table 3). There was no significant difference in DFS and OS according to level of FoxP3-TSDR demethylation.

**Table 2:** Univariate and multivariate analyses on DFS.

Factor	N	Univariate		Multivariate	
		HR (95% CI)	*p*-value	HR (95% CI)	*p*-value
Peripheral ImmunoCRIT					
Low	230	1		1	
High	192	1.5 (1.0–2.2)	0.031	1.5 (1.0–2.3)	0.060
Age					
<70	342	1		-	
≥70	80	1.8 (1.2–2.7)	0.006	-	
Lymphatic invasion					
Absent	127	1		-	
Present	293	1.9 (1.2–3.1)	0.005	-	
Vascular invasion					
Absent	352	1		1	
Present	68	3.6 (2.4–5.3)	<0.001	2.5 (1.6–3.8)	<0.001
Perineural invasion					
Absent	144	1		1	
Present	275	3.0 (1.8–4.9)	<0.001	1.7 (1.0–3.0)	0.043
Stage (AJCC 7th)					
II	203	1		1	
III	219	3.4 (2.2–5.2)	<0.001	2.5 (1.6–3.8)	<0.001
Neutrophil to lymphocyte ratio					
Low (<2.5)	331	1		-	
High (≥2.5)	82	1.7 (1.1–2.5)	0.017	-	
Prognostic nutritional index					
High (≥54)	189	1		1	
Low (<54)	199	2.2 (1.5–3.3)	<0.001	1.5 (1.0–2.3)	0.065

Multivariate analysis by Cox proportional hazard model. HR, hazard ratio; DFS, disease-free survival; CI, confidence interval.

**Table 3:** Univariate and multivariate analyses on OS.

Factor	N	Univariate		Multivariate	
		HR (95% CI)	*p*-value	HR (95% CI)	*p*-value
Peripheral ImmunoCRIT					
Low	230	1		1	
High	192	1.8 (1.1–3.0)	0.020	1.9 (1.2–2.9)	0.005
Age					
<70	342	1		-	
≥70	80	2.1 (1.3–3.2)	0.002	-	
Lymphatic invasion					
Absent	127	1		-	
Present	293	1.7 (1.1–2.7)	0.031	-	
Vascular invasion					
Absent	352	1		1	
Present	68	3.5 (2.3–5.2)	<0.001	2.9 (1.9–4.5)	<0.001
Perineural invasion					
Absent	144	1		1	
Present	275	2.8 (1.7–4.8)	<0.001	1.8 (1.0–3.1)	0.039
Stage (AJCC 7th)					
II	203	1		1	
III	219	2.8 (1.8–4.3)	<0.001	1.9 (1.2–3.2)	0.010
Neutrophil to lymphocyte ratio					
Low (<2.5)	331	1		-	
High (≥2.5)	82	1.8 (1.2–2.8)	0.010	-	
Prognostic nutritional index					
High (≥54)	189	1		-	
Low (<54)	199	2.0 (1.3–3.1)	0.001	-	

Multivariate analysis by Cox proportional hazard model. HR, hazard ratio; DFS, disease-free survival; CI, confidence interval.

### Peripheral ImmunoCRIT and immune cells in the TIME

Table 4 shows the association between peripheral ImmunoCRIT and immune cell infiltration in the TIME. CD3+ cell counts within the tumor were lower in low ImmunoCRIT than those in high ImmunoCRIT (mean CD3+ cell count in central tumor area: 201.2/mm^2^ *vs*. 172.2/mm^2^, *p* = 0.029). CD4+ cell counts were also lower in low ImmunoCRIT than those in high ImmunoCRIT (mean CD4+ in central tumor area: 56.5/mm^2^ *vs*. 43.5/mm^2^, *p* = 0.007). No significant relationship was observed between the peripheral ImmunoCRIT and CD45RO, arginase, CD68+, CD163+, CD8+, and FoxP3+ immune cell infiltration in the TIME.

**Table 4:** Association between peripheral ImmunoCRIT and tumor immune microenvironment in resected gastric cancer.

		Peripheral ImmunoCRIT		*p*-value
		High	Low	
CD45RO, cells/mm^2^	Tumor central	126.8 (103.5)	125.5 (90.4)	0.901
	Tumor peripheral	81.8 (77.3)	92.6 (82.6)	0.195
Arginase, pixel/mm^2^	Tumor central	139726.4 (721655.8)	112446.4 (204730.4)	0.631
	Tumor peripheral	63056.7 (136848.4)	49222.6 (112344.7)	0.334
CD68, pixel/mm^2^	Tumor central	402842.4 (317123.3)	378560.9 (313045.1)	0.459
	Tumor peripheral	271870.7 (235358.5)	298407.6 (260537.8)	0.301
CD163, pixel/mm^2^	Tumor central	156599.0 (212138.9)	164302.7 (189831.0)	0.714
	Tumor peripheral	183522.1 (224505.5)	231384.1 (309892.7)	0.084
CD3, cells/mm^2^	Tumor central	201.2 (146.0)	172.2 (110.2)	0.029
	Tumor peripheral	147.6 (132.8)	152.5 (132.0)	0.720
CD4, cells/mm^2^	Tumor central	56.5 (48.4)	43.5 (42.3)	0.007
	Tumor peripheral	36.8 (38.4)	37.0 (33.9)	0.952
CD8, cells/mm^2^	Tumor central	140.5 (104.9)	136.2 (100.8)	0.686
	Tumor peripheral	95.8 (83.3)	110.8 (87.2)	0.091
Foxp3, cells/mm^2^	Tumor central	136.8 (110.9)	146.9 (106.0)	0.369
	Tumor peripheral	95.5 (113.5)	120.3 (136.4)	0.060

SD, Standard deviation. Data are presented as mean (standard deviation).

We also evaluated the relationship between peripheral blood FoxP3 (demethylated FoxP3-TSDR) and tumor FoxP3 expression, and there was no significant correlation (tumor central Foxp3, pearson’s coefficient -0.35, *p* = 0.497; tumor peripheral area Foxp3, pearson coefficient -0.41, *p* = 0.432). Peripheral blood CD3 (unmethylated CD3G) and tumor CD3 expression did not show significant correlation (tumor central CD3, pearson’s coefficient -0.024, *p* = 0.643; tumor peripheral area CD3, pearson coefficient -0.009, *p* = 0.860).

## Discussion

In this study, the pTregs-to-tTL ratio known as ImmunoCRIT was measured by epigenetic methylation assay using pyrosequencing from long-term stored peripheral blood as a liquid immune biomarker. High ImmunoCRIT was associated with higher CD3+ and CD4+ cell density in the TIME as compared with low ImmunoCRIT. Patients with high ImmunoCRIT had shorter OS than those with low ImmunoCRIT, regardless of systemic inflammatory markers such as NLR and PNI.

Since immunotherapy became the center of attention in cancer treatment, many studies have focused on the characteristics of immune cells constituting the TME [16–18]. Apart from the local TIME, immune cell constitution in the peripheral blood may be correlated with prognosis and response to immunotherapy, as many cytokines and inflammatory cells migrate from blood to local tissues via systemic circulation. Systemic inflammation has already been known to be associated with the prognosis of cancer patients, and systemic inflammation markers such as NLR and PNI are correlated with prognosis and survival rate in many solid tumors [4, 19, 20]. Thus, local and systemic immunity are both an important factor in response to chemotherapy and immunotherapy. TIME is a part of the host immunity; therefore, identifying peripheral blood markers that can predict the local TIME status and response to chemotherapy and immunotherapy may be possible. In this study, peripheral blood ImmunoCRIT determined by epigenetic methylation assay was correlated with specific immune cell infiltration in resected gastric tumors. High peripheral ImmunoCRIT was significantly associated with higher CD3+ and CD4+ cell counts within the central tumor area. Additionally, high ImmunoCRIT was an independent prognostic factor for shorter OS in resected gastric cancer, even after adjusting for systemic inflammation markers such as NLR and PNI. NLR was not an independent prognostic factor for neither PFS nor OS in multivariate analysis including the ImmunoCRIT status as a covariant. This suggests that the composition of peripheral blood lymphocytes, not just the NLR, has biological significance and prognostic value in patients with resected gastric cancer.

The prognostic value of FoxP3+ Tregs in ‘tumor microenvironment’ remains controversial. Tumor-infiltrating FoxP3+ Tregs were associated with better survival in colorectal, head and neck, and esophageal cancers [21], and higher tumor-infiltrating Foxp3+ cells were associated with better OS in patients with resected gastric cancer [22]. In contrast, in a meta-analysis encompassing 17 cancer types and 15,512 cancer patients, tumor-infiltrating FoxP3+ Tregs was associated with poor OS in the majority of solid tumors including gastric cancer [21], and Kim et al. also reported that a high Foxp3+ Tregs/CD4 ratio in tumor was an unfavorable prognostic factor for OS in gastric cancer [23]. Shen et al. [24] and Perrone et al [25] also reported unfavorable prognosis with increased intratumoral Tregs. In our study, high ImmunoCRIT showed worse prognosis compared to low ImmunoCRIT, supporting the unfavorable prognostic role of peripheral Foxp3+ Tregs in gastric cancer.

Data on “peripheral blood” Tregs and their association with the prognosis of cancer patients are also limited. The peripheral ImmunoCRIT of the healthy general population was an independent risk factor for lung, colorectal, and estrogen receptor negative breast cancer development in a case-cohort study [10]. In this study, high ImmunoCRIT showed worse prognosis and low ImmunoCRIT showed better prognosis. In another study focusing on patients with untreated non-small cell lung cancer, unlike the naïve or effector Tregs, high levels of terminal effector Tregs were correlated with improved clinical outcomes, suggesting that the prognosis may differ depending on the subtype of Tregs [26]. Since no Tregs subtype analysis was performed in this study, it is not known whether the low ImmunoCRIT group had a better prognosis due to the large proportion of terminal effector Tregs. The association of peripheral blood Tregs or ImmunoCRIT and the specific subtypes of Tregs and the prognosis of cancer patients need further investigation.

This study has some limitations. First, data splitting for training and test cohort experiment for internal validation was not performed. Also, external validation was not carried out due to unavailability of separate cohort with long term follow-up and available blood samples for this methylation analyses. Although our cohort was relatively large to demonstrate the prognostic value of ImmunoCRIT, further investigation and validation in other population is required. Second, our study showed statistically significant association with ImmunoCRIT and survival in gastric cancer patients, but the significance level is not extremely strong considering that multiple comparisons were done for correlated outcomes. Furthermore, the clinical significance in patients who received immunotherapies should be evaluated. Third, we found that ImmunoCRIT was associated with CD3+ and CD4+ cells in TIME. However, the underlying mechanism explaining the association of systemic immune cell components with CD3+ and CD4+ cells in TIME was not evaluated and requires the further investigation.

In conclusion, our results suggest that ImmunoCRIT determined by epigenetic methylation analysis is correlated with specific immune cell infiltration within the TME in resected gastric tumors and provides a prognostic information in patients with gastric cancer. ImmunoCRIT could be an easily obtained potential biomarker for prognosis in future immunotherapeutic treatment strategies.

## Supporting information

S1 Method (DOCX)

S1 Fig Two pyrograms analyzing 4 or 6 CpG sites of the FOXP3-TSDR and CD3D/CD3G genes. (DOCX)

S2 Fig Epigenetic patterns of sorted immune cells assessed by bisulfite pyrosequencing. (DOCX)

S1 Table Targets CpG islands and the primers for pyrosequencing. (DOCX)

S2 Table Baseline characteristics according to Foxp3-TSDR demethylation. (DOCX)

S1 Data (XLSX)
