# Supplementary material for: Correlation between tumor infiltrating immune cells and peripheral regulatory T cell determined using methylation analyses and its prognostic significance in resected gastric cancer
Source: PLoS One. 2021 Jun 4;16(6):e0252480. doi: 10.1371/journal.pone.0252480 (PMC8177409; doi:10.1371/journal.pone.0252480)
Supplement: S1 Method — (DOCX) [file pone.0252480.s001.docx]

**S1 Method**

**Sodium bisulfite modification method**

Bisulfite-modified gDNA was prepared using the EZ DNA Methylation-Lighting^TM^ kit (Zymo Research, CA, USA) according to the manufacturer’s instructions. The bisulfite reaction was carried out on 200 ng gDNA, and the reaction volume was adjusted to 20 ul with sterile water and added with 130 ul of CT conversion reagent. Sample tubes were placed in a thermal cycler (MJ Research, Quebec, Canada) following these steps: 8 min at 98°C, 60 min at 54°C, and stored at 4°C for up to 20 h. The DNA was purified using a reagent contained in EZ DNA Methylation- Lighting^TM^ kit (Zymo Research, CA, USA). The converted samples were added into Zymo-Spin IC^TM^ Column containing 600 ul of the M-Binding Buffer and mixed by inverting the column several times. The column was centrifuged at full speed for 30 s, and the flow through discarded. The column was washed by adding 200 ul of M-Wash Buffer and spun at full speed and then 200 ul of M-Desulphonation Buffer was added to the column and let stand at room temperature (20–30°C) for 15–20 min. After incubation, the column was centrifuged at full speed for 30 s and then washed by adding 200 ul of M-Wash Buffer and spun at full speed (repeat this step). The converted gDNA was eluted by adding 20 ul of M-Elution Buffer into the column and spun. DNA samples were finally stored at -20°C until further use.

**Pyrosequencing analysis method**

Each primer was designed based on those target regions using the Pyrosequencing Assay Design Software v2.0 (Qiagen, Hilden, Germany). Polymerase chain reaction (PCR) was carried out in a volume of 20 ul with ≥20 ng converted into gDNA, PCR premixture (Enzynomics, Korea), 1 ul of 10 pmole/ul Primer-S, and 1 ul of 10 pmole/ul biotinylated-Primer-As. The amplification was carried out according to the general guidelines suggested by Pyrosequencing: denaturating at 95°C for 10 min, followed by 45 cycles at 95°C for 30 s, at 56/60°C for 30 s, at 72°C for 30 s, and a final extension at 72°C for 5 min. PCR (2 ul) was confirmed by electrophoresis in a 2% Agarose gel and visualized using the ethidium bromide staining. The ssDNA template was prepared from 16–18 ul biotinylated PCR product using streptavidin Sepharose^®^ HP beads (Amersham Biosciences, Sweden) following the PSQ 96 sample preparation guide using multichannel pipets. Fifteen picomoles of the respective sequencing primer were added for analysis. Sequencing was performed using a PyroMark ID system with the Pyro Gold reagents kit (Qiagen, Hilden, Germany) according to the manufacturer’s instruction without further optimization.

**Tissue microarray construction and antibody information**

Tissue microarrays were were generated as follows. Tissue samples from surgical specimens were fixed in 10 % buffered formalin for 24–48 h and then embedded in paraffin. Representative cores (2 mm in diameter) were isolated from individual paraffin blocks and arranged in new tissue array blocks with use of a trephine apparatus (Superbiochips Laboratories, Seoul, Korea). The patients included in the study had tumors occupying more than 10 % of the core area. Nonneoplastic gastric mucosa specimens were also incorporated into each of the array blocks, and the tissue array blocks contained up to 60 cores.

Arrays were stained with the following primary antibodies: rabbit polyclonal anti-CD3 (1:100 dilution, Dako, Glostrup, Denmark); rabbit monoclonal anti-CD4, clone SP35 (undiluted, Ventana Medical Systems, Tucson, AZ, USA); mouse monoclonal anti-CD8, clone C8/144B (undiluted, Dako, Carpinteria, CA, USA); mouse anti-FoxP3, clone 236A/E7 (1:50 dilution, Abcam, Cambridge, UK); mouse anti-CD45RO, clone UCLH-1 (1:100 dilution, Cell Marque, Rocklin, CA, USA); rabbit anti-arginase (1:100 dilution, Thermo Fischer Scientific, Waltham, MA, USA); mouse anti-CD68, clone PG-M1 (1:100 dilution, Dako); and mouse anti-CD163, clone 10D6 (1:100 dilution, Novocastra, Newcastle upon Tyne, UK).

**Image analysis method**

All immunostained slides were scanned at 200× magnification using an Aperio ScanScope CS2 instrument (Aperio Technologies, Vista, CA, USA) and analyzed with computerized image analysis algorithms using ImageScope (version 12.0.0.5039, Aperio Technologies). Cells positive for CD3, CD4, CD8, FoxP3, and CD45RO were quantified using the Nuclear V9 algorithm, and positive cell densities for each immune cell subset were defined as the number of positive cells per mm^2^. CD68+, CD168+, and arginase+ immune cells were assessed using the Positive Pixel Count V9 algorithm, and positive cell densities for these markers were calculated as positive pixels per mm^2^.
