# Supplementary figures and images for: Correlation between tumor infiltrating immune cells and peripheral regulatory T cell determined using methylation analyses and its prognostic significance in resected gastric cancer
Source: PLoS One. 2021 Jun 4;16(6):e0252480. doi: 10.1371/journal.pone.0252480 (PMC8177409; doi:10.1371/journal.pone.0252480)

**S1 Fig. Two pyrograms analyzing 4 or 6 CpG sites of the FOXP3-TSDR and CD3D/CD3G genes**

**
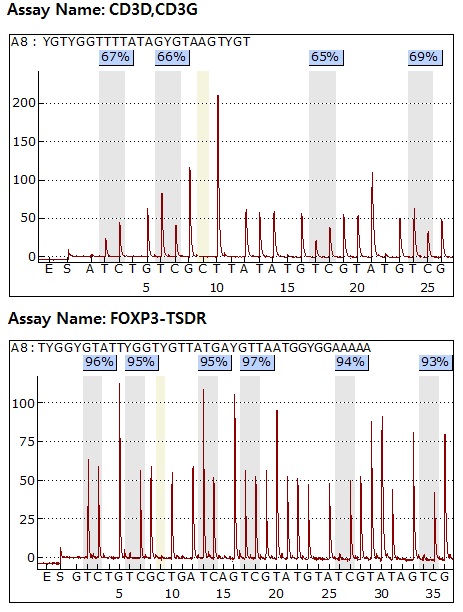
**

Supplement: S1 Fig — (DOCX) [file pone.0252480.s002.docx]
