# Supplementary material for: Correlation between tumor infiltrating immune cells and peripheral regulatory T cell determined using methylation analyses and its prognostic significance in resected gastric cancer
Source: PLoS One. 2021 Jun 4;16(6):e0252480. doi: 10.1371/journal.pone.0252480 (PMC8177409; doi:10.1371/journal.pone.0252480)
Supplement: S2 Fig — (DOCX) [file pone.0252480.s003.docx]

**S2 Fig. Epigenetic patterns of sorted immune cells assessed by bisulfite pyrosequencing**
