# Supplementary material for: Correlation between tumor infiltrating immune cells and peripheral regulatory T cell determined using methylation analyses and its prognostic significance in resected gastric cancer
Source: PLoS One. 2021 Jun 4;16(6):e0252480. doi: 10.1371/journal.pone.0252480 (PMC8177409; doi:10.1371/journal.pone.0252480)
Supplement: S1 Table — (DOCX) [file pone.0252480.s004.docx]

**S1 Table. Targets CpG islands and the primers for pyrosequencing**

| Gene | Primer | | Size(bp) |
| --- | --- | --- | --- |
| *FOXP3-TSDR* | Forward | 5’- GTTAAGTTTGTTGTAGGATAGGGTAGT -3’ | 181 |
|  | Biotinylated-reverse | 5’- AATCTACATCTAAACCCTATTATCACA -3’ |  |
|  | Sequencing primer | 5’- GTGGTGTAGATGAAGT -3’ |  |
| *CD3D/CD3G* | Forward | 5’- TTGGTAGAGAATATGGAAAAGGTG -3’ | 213 |
|  | Biotinylated-reverse | 5’- ACTACTAAATCTAATATCCCTAACTACCTA -3’ |  |
|  | Sequencing primer | 5’- GAAGTTTTTTGAGAGAAGT -3’ |  |
