# Supplementary material for: Correlation between tumor infiltrating immune cells and peripheral regulatory T cell determined using methylation analyses and its prognostic significance in resected gastric cancer
Source: PLoS One. 2021 Jun 4;16(6):e0252480. doi: 10.1371/journal.pone.0252480 (PMC8177409; doi:10.1371/journal.pone.0252480)
Supplement: S2 Table — (DOCX) [file pone.0252480.s005.docx]

**S2 Table. Baseline characteristics according to Foxp3-TSDR demethylation**

|  |  | **Demethylated Foxp3-TSDR** | |  |
| --- | --- | --- | --- | --- |
| **Variables** |  | **<6.7% (N=212)** | **≥6.7% (N=210)** | ***p-*value** |
| Age (years) | < 70 | 160 (46.8) | 182 (53.2) | 0.003 |
|  | ≥ 70 | 52 (65.0) | 28 (35.0) |  |
| Sex | Male | 211 (77.0) | 63 (23.0) | <0.001 |
|  | Female | 1 (0.7) | 147 (99.3) |  |
| Lauren classification | Intestinal | 100 (64.9) | 54 (35.1) | <0.001 |
|  | Diffuse | 88 (38.6) | 140 (61.4) |  |
|  | Mixed | 22 (61.1) | 14 (38.9) |  |
| Stage by AJCC 7th | II | 109 (53.7) | 94 (46.3) | 0.171 |
|  | III | 103 (47.0) | 116 (53.0) |  |
| NLR | Low (<2.5) | 161 (48.6) | 170 (51.4) | 0.159 |
|  | High (≥2.5) | 47 (57.3) | 35 (42.7) |  |
| PNI | High (≥54) | 86 (45.5) | 103 (54.5) | 0.104 |
|  | Low (<54) | 107 (53.8) | 92 (46.2) |  |
| Adjuvant chemotherapy | No adjuvant | 0 (0) | 1 (100) | 0.763 |
|  | TS-1 | 183 (50.7) | 178 (49.3) |  |
|  | Capecitabine + oxaliplatin | 19 (47.5) | 21 (52.5) |  |
|  | Fluorouracil + cisplatin | 10 (50.0) | 10 (50.0) |  |

AJCC, American Joint Committee on Cancer; NLR, neutrophil to lymphocyte ratio; PNI, prognostic nutritional index; AJCC, American Joint Committee on Cancer.
